# Investigation of the effect of rifampicin resistance and risk factors on recovery rates after DAIR procedure in patients with prosthetic joint infection

**DOI:** 10.1186/s13018-023-04091-y

**Published:** 2023-08-21

**Authors:** Gergely Krizsán, Imre Sallai, Dániel Sándor Veres, Gyula Prinz, Máté Kovács, Gábor Skaliczki

**Affiliations:** 1 Department of Orthopaedics, Semmelweis University, Budapest, Hungary; 2 Department of Biophysics and Radiation Biology, Semmelweis University, Budapest, Hungary

**Keywords:** Prosthetic joint infection, DAIR, Recovery rates, Rifampicin resistance, Risk factors

## Abstract

**Background:**

Rifampicin plays a key role in the management of prosthetic joint infections (PJIs), however, the emergence of rifampicin resistance is associated with less favourable clinical outcomes. The purpose of this study was to investigate the impact of rifampicin resistance and other patient-related factors on recovery rates among patients with PJI undergoing debridement, antibiotics and implant retention (DAIR).

**Methods:**

We reviewed medical records and microbiology reports of 67 patients (37 males and 30 females) undergoing DAIR due to PJI between 2014 and 2021. Patient-related factors, co-morbidities and microbiological reports were collected and reviewed. Forty-four patients had hip, 21 had knee, 1 had shoulder and 1 had elbow joint infection. Obtained data were statistically analysed with a logistic regression model.

**Results:**

Rifampicin-sensitive organism was isolated in 47 cases. Recovery rate was 72.3% in the sensitive and 76.9% in the resistant group. We found no significant effect of rifampicin resistance on the probability of recovery. Age and diabetes mellitus showed negative clinical impact on recovery. *Staphylococcus aureus* and coagulase-negative Staphylococci were predominant in the rifampicin-sensitive (66.6% of the isolates) and Gram-negative rods in the resistant group (65.2%).

**Conclusions:**

Based on our results, higher age and diabetes mellitus may have a clinically relevant negative impact on clinical outcome, however, this effect was not statistically significant. This may be due to the limited number of patients included in this study. We observed no clinically relevant effect of rifampicin-resistance, sex and body mass index (BMI) on recovery rates among patients undergoing DAIR due to PJI.

## Background

The incidence of prosthetic joint infections (PJIs) has shown increasing trend worldwide which can be attributed to several factors including higher number of arthroplasties and the ageing population [1]. *Staphylococcus aureus* and coagulase-negative Staphylococci (CNS) are the most frequent causative agents, however, further Gram-positive and Gram-negative bacteria as well as fungal pathogens can also be involved, potentially resulting in polymicrobial infections [2, 3].

Both Gram-positive and Gram-negative microorganisms have the ability to form biofilms on the surface of prosthetic materials, particularly Staphylococci and *Pseudomonas aeruginosa* [4, 5]. This should always be considered in the treatment of PJIs, hence rifampicin is frequently used with other antibiotics in such cases. Combination is essential as monotherapy can rapidly lead to rifampicin resistance [6, 7]. However, resistance may develop even when rifampicin is used in combination, especially in the presence of certain predisposing factors including postoperative drainage, fistula, open wound and abscess [8]. The aim of the treatment is to eradicate pathogens, provide bactericidal as well as anti-biofilm activity and reduce the risk of resistance development [9].

Difficult-to-treat (DTT) pathogens have the potential to develop resistance against antibiotics and produce biofilm resulting in difficult eradication. Microbiological diagnosis can also be challenging for some species, eg. *Cutibacterium acnes* and Finegoldia spp. A study found that as high as 58% of the patients had PJI caused by DTT organisms with less favourable prognosis and the need of prolonged treatment [3].

The purpose of this study was to investigate the effect of rifampicin resistance and patient-related factors on recovery rates in patients with PJI undergoing DAIR procedure (Debridement, Antibiotics and Implant Retention). Co-morbidities and other medical conditions, orthopaedic factors including revision before DAIR procedure, mobile element exchange, preoperative score systems as well as antibiotic treatment regimes were also reviewed.

## Methods

### Study participants

Our study was approved by the Ethics Committee of Semmelweis University and carried out in accordance with the Declaration of Helsinki. All patients gave their informed consent and were anonymised. We reviewed the medical records of 67 patients (37 males and 30 females) admitted to our department (Department of Orthopaedics, Semmelweis University, Budapest, Hungary) undergoing DAIR procedure due to early onset PJI (starting within 6 weeks after the index surgery according to the International Consensus Meeting on Musculoskeletal Infections meeting criteria [10]) between 2014 and 2021. Patients who had revisions with implant removal were not included in this study. The mean age was 68.4 years (standard deviation (SD) = 15.8 years), and the mean body mass index was 30.5 kg/m^2^ (SD = 5.87 kg/m^2^), respectively. 11 patients (16.4%) had diabetes mellitus (DM): 2 had Type 1 and 9 had Type 2 DM. 44 patients had hip, 21 had knee, 1 had shoulder and 1 had elbow joint infection. The end date of the follow up period was 31 December, 2022.

Past medical history, risk factors, co-morbidities and clinical details were collected and analysed. In our cross-sectional study, patients were divided into two groups according to rifampicin sensitivity results of the organism(s) causing PJI. After statistical description of our data, we compared recovery rates among patients within the two groups and investigated the effect of patient-related factors including the affected joint, previous trauma, treatment duration, antibiotic regime(s), administration of jet lavage, exchange of mobile elements and revision before DAIR procedure. Sex, age, comorbidities (including diabetes mellitus, chronic obstructive pulmonary disease [COPD], rheumatoid arthritis [RA], chronic renal failure [CRF], liver cirrhosis, thyroid diseases, hypertension and coagulation abnormalities), ASA score and BMI were reviewed. CRIME80 and KLIC scores were calculated preoperatively to estimate the risk of failure of DAIR procedure and the recurrence of PJI. Patients are followed up 6 weeks, 3 months and 6 months after surgery and on a yearly basis thereafter. However, if clinically indicated, patients are re-assessed more frequently. Recovery is considered in patients with no clinical, radiological or laboratorical signs of infection after a follow-up period of two years.

### Microbiology reports

Clinical specimens were processed in the Clinical Microbiological Diagnostic Laboratory (Institute of Laboratory Medicine, Semmelweis University, Budapest, Hungary) with conventional methods including microscopy, culture and antibiotic sensitivity testing (disk diffusion and E-tests according to the European Committee on Antimicrobial Susceptibility Testing [EUCAST] guidelines). Microbiology reports (including antibiograms) were collected, and clinical significance of each isolate was assessed. The most frequent types of specimens were punctures and aspirates (either cultured directly or incubated in blood culture bottles) as well as intraoperative deep wound swabs. We also reviewed antibiotic regimes used to treat PJIs and whether patients had received rifampicin prior to their current orthopaedic infection. Development of rifampicin-resistance during treatment and polymicrobial infections were also investigated.

### Statistical analysis

The statistical analysis was performed by using the R software [11] and its ggplot2 package for figures [12]. After describing data, a logistic regression model was fitted: we used recovery rate as the outcome and rifampicin resistance as the explanatory variable. The effect was controlled for sex, age, BMI and DM as possible confounders. The interaction between rifampicin resistance and age was assessed, however, as it shows no relevant and significant effect, it is not included in the final model. Decisions were made on null-hypothesis using 5% as significance level. No multiplicity correction was made.

## Results

### Recovery rates

Selected data of the study population are summarised in Table 1. 47 (70.1%) patients had rifampicin sensitive and 13 (19.4%) patients had resistant isolate. The overall recovery rate was 74.6% (50 out of 67 patients), 72.3% among patients within the rifampicin-sensitive group and 76.9% in the resistant group, respectively. 15 patients (22.4%) had therapeutic failure. Significant pathogens were isolated in 60 out of 67 cases.

**Table 1:** Summary of study population data used for statistical analysis

	Not recovered (N = 17)	Recovered (N = 50)	Overall (N = 67)
Rifampicin resistance			
Sensitive	13 (76.5%)	34 (68.0%)	47 (70.1%)
Resistant	3 (17.6%)	10 (20.0%)	13 (19.4%)
No data available	1 (5.9%)	6 (12.0%)	7 (10.4%)
Sex			
Male	10 (58.8%)	27 (54.0%)	37 (55.2%)
Female	7 (41.2%)	23 (46.0%)	30 (44.8%)
Age [years]			
Mean (SD)	69.4 (15.8)	68.1 (18.0)	68.4 (15.8)
Median (IQR)	73.7 (13.2)	72.6 (14.8)	72.8 (14.4)
Min, Max	26.9, 86.7	28.5, 86.7	26.9, 86.7
Diabetes mellitus			
Non-diabetic	13 (76.5%)	43 (86.0%)	56 (83.6%)
Diabetic	4 (23.5%)	7 (14.0%)	11 (16.4%)
BMI [kg/m^2^]			
Mean (SD)	30.1 (5.21)	30.7 (6.12)	30.5 (5.87)
Median (IQR)	27.6 (4.99)	29.4 (8.20)	29.0 (7.85)
Min, Max	24.3, 42.4	18.7, 46.9	18.7, 46.9
No data available	0 (0%)	1 (2.0%)	1 (1.5%)

First, we investigated the effect of selected variables such as rifampicin resistance, sex, age, DM and BMI in a multivariate regression model. According to our analysis, we found no statistically significant effect of these factors on recovery rates (Table 2). We also reviewed further risk factors including hypertension, ASA score, C-reactive protein (CRP), COPD, liver cirrhosis, CRF, haematology disorders, RA and whether implant included cement; however, we could not perform a statistical inferential analysis due to the low number of patients with certain risk factors.

**Table 2:** Effect estimates of risk factors with its 95% confidence interval based on a regression model

Predictors	Odds ratio (recovered vs. not recovered)	95% confidence interval	*p* value
(intercept)	7.5069	0.1021–803.8825	0.3697
Rifampicin resistance: Resistant	1.2372	0.3046–6.3043	0.7766
Age [years]	0.9892	0.9478–1.0259	0.5801
Sex Male	0.7513	0.2048–2.5711	0.6534
BMI [kg/m^2^]	1.0003	0.8985–1.1203	0.9956
Diabetes mellitus: Diabetic	0.3948	0.0876–1.8473	0.2206

**Fig. 1:**
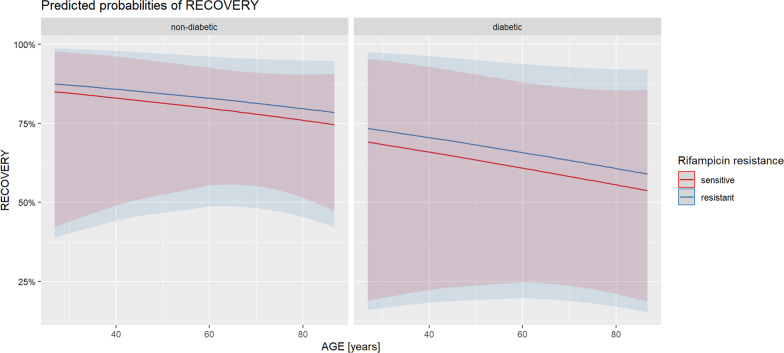
Predicted recovery probability based on a regression model (for female, at mean BMI with 95% confidence interval)

11 patients (16.4%) had diabetes mellitus: 9 had Type 2 and 2 had Type 1. We can assume a clinically important impact of age on recovery rates both in the rifampicin-sensitive and in the resistant group (Fig. 1), although it may prove difficult to assess the significance of this effect due to the relatively low number of patients and possible unknown confounders. We also observed the negative clinical effect of diabetes mellitus on recovery rates in our cohort. However, further data are required to confirm these findings.

The mean age was 67.4 years, and 11 (23.4%) patients were more than 80 years old in the rifampicin sensitive group. However, in the resistant group, the mean age was 70.1 years and 5 (38.5%) patients were over 80 years of age. Altogether 50 patients (74.6%) recovered and 17 (25.4%) had treatment failure. 72.3% of patients recovered in the rifampicin-sensitive group and 76.9% in the resistant group. Among recovered patients, mean age was lower in the sensitive group (65.6 vs 73.7 years), however, the opposite was observed among patients without recovery (71.9 vs 57.8 years), respectively.

25.5% of the patients in the sensitive group had revision before DAIR procedure and 38.5% in the resistant group. However, there was no clinically relevant difference in the rates between the two groups after DAIR (19.1% and 15.4%, respectively). We also investigated how revision before DAIR influenced recovery rates. There were 12 revisions in the rifampicin-sensitive group with 66.6% recovery rate and 5 revisions in the resistant group with 80.0% recovery. Without previous revision, the recovery rates were 74.3% and 75.0%, respectively.

The exchange of mobile elements was also reviewed: 16 patients had head exchange, 19 had insert exchange, 2 had both and 30 had none. Altogether 55.3% of the patients had exchange in the sensitive and 46.2% in the resistant group. Insert exchange was less, whereas head exchange and no exchange were more predominant in the resistant group. Insert exchange resulted in higher recovery rate and by the exchange of more components an even higher rate was achieved in the rifampicin-sensitive group. When no exchange was performed, recovery rates were identical in the two groups (71.4%, respectively).

Recovery rates were compared among patients with CRIME80 score ≥ 3 (19 cases). 100% recovery was seen in the resistant and 66.7% in the sensitive group. Average score of recovered patients in the sensitive group was 1.65, whereas it was 2.1 in the resistant group. Nineteen patients had higher than 4.5 KLIC score. 2 out of 2 patients recovered in the resistant group and recovery rate was 70.6% in the sensitive group. Average score of recovered patients in the sensitive group was 2.91, whereas it was 2.85 in the resistant group. 92.3% of the patients had KLIC score in the 2–3.5 range in the resistant group. Although the same range was predominant in the sensitive group (42.6%), the distribution was more balanced.

### Microbiological background

We reviewed the microbiology reports of all patients included in this study. Whenever not tested, *Cutibacterium* (formerly *Propionibacterium) acnes, Finegoldia magna* and Streptococci were categorised as rifampicin-sensitive, whereas Enterococci, *Enterobacterales* and *P. aeruginosa* as rifampicin-resistant according to their natural resistance profile and expert rules.

**Fig. 2:**
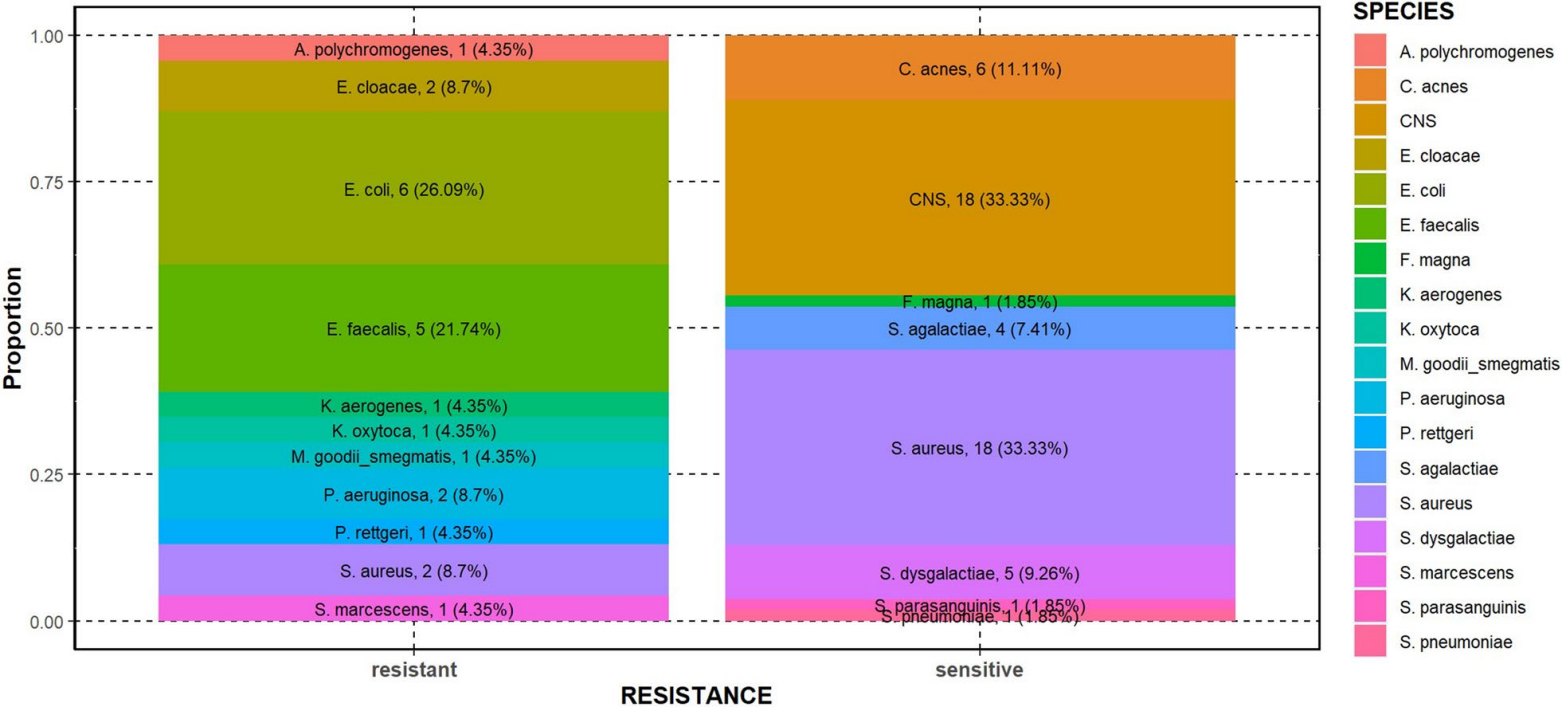
Pathogen distribution of rifampicin-resistant and -sensitive isolates (count, percentage)

Fourty-seven out of 67 patients (70.1%) had PJI caused by rifampicin-sensitive and 13 (19.4%) by rifampicin-resistant organism. We found that rifampicin-sensitive organism was isolated in 77.3% among recovered patients and 81.3% in patients with treatment failure. Staphylococcus spp. were predominant in the rifampicin-sensitive group (66.7% of the isolates) including 18 *S. aureus* and 18 CNS isolates. Other pathogens from the sensitive group included Streptococcus spp. (11 isolates, with the predominance of *S. agalactiae* and *S. dysgalactiae*) and Gram-positive rods (6 *C. acnes*), respectively. However, the pathogen distribution was significantly different in the resistant group: Staphylococci were less prevalent (2 *S. aureus* isolates) and Gram-negative rods (12 *Enterobacterales* and 2 *P. aeruginosa*) as well as Enterococcus spp. (5 *E. faecalis*) were predominant. Among *Enterobacterales*, *Escherichia coli* was the most frequent species. A rifampicin-resistant strain of *Arthrobacter polychromogenes* and *Mycobacterium goodii/smegmatis* was also isolated (Fig. 2). 3 out of 20 *S. aureus* isolates were methicillin-resistant (MRSA). Development of rifampicin resistance was not observed in our cohort and none of the patients had received previous rifampicin treatment. Three patients had polymicrobial infection in the sensitive group and four in the resistant group. Of note, Gram-positive bacteria were involved in all these cases and 70.0% of the pathogens were resistant to rifampicin.

Antibiotic regimes were also reviewed in our study. Rifampicin administration was documented in 34 cases in combination with other antibiotics depending on microbiology results and patient-related factors. Of note, rifampicin was used in monotherapy in two cases. Combination partners included ciprofloxacin, levofloxacin, sulfamethoxazole/trimethoprim, doxycycline and amoxicillin. In one case, a triple therapy of amoxicillin, ciprofloxacin and rifampicin was established. When comparing antibiotic regimes, ciprofloxacin was the combination partner when the highest number of rifampicin-resistant organisms were isolated (30.8%). The most frequent combinations in the sensitive group were sulfamethoxazole/trimethoprim + rifampicin (19.1%) and levofloxacin + rifampicin (17.0%).

It is also interesting to note the recovery rates in relation to significant isolates: 75.0% of the patients recovered when rifampicin resistant *S. aureus* was isolated and 56.1% when the isolate was sensitive to rifampicin. 75.0% of the patients recovered in the sensitive and 66.7% in the resistant group if CNS was isolated. All the three patients with MRSA infection had treatment failure, whereas three out of four patients with *E. coli* and all the five patients with Cutibacteria recovered. 81.8% of the patients with streptococcal infection showed recovery.

## Discussion

DAIR procedure is most efficient in the early postoperative period (< 6 weeks) and in acute haematogenous infections. The nonmobile elements of the prosthesis are left in situ during surgery. Debridement includes exploration of the joint, removal of necrotic and infected tissues, the use of high volume washing fluid and the exchange of mobile components. The surgical procedure is followed by prolonged antibiotic treatment [13]. The aim of the procedure is to save the prosthesis by eradicating the infection and removing the biofilm. It is recommended to perform open surgery as arthroscopic synovectomy is unsatisfactory [14, 15]. However, DAIR procedure cannot be used for the treatment of late PJIs as compact and mature biofilm can only eradicated by the exchange of prosthesis [16]. There are different recommendations regarding antibiotic route and duration, however, all regimes include prolonged intravenous treatment followed by oral antibiotics. The duration of intravenous antibiotic therapy ranges from 2 to 6 weeks and the total duration of treatment is 12 weeks in most of the cases, however it can be as long as 24 weeks if knee joint is involved. Moreover, treatment regime is also determined by the type and outcome of surgical procedure(s). Rifampicin has essential role in the management of infections with biofilm formation [17]. The dose of rifampicin is 300–600 mg twice daily *per os*. Liver function tests should be reviewed before starting treatment and followed up on a regular basis. Also, drug interactions must be carefully assessed when antibiotic therapy is being planned.

Postoperative wound discharge can be a sign as well as a predisposing factor of PJI. Ongoing discharge may act as a basis of infection which can spread to deeper tissues and reach the surface of implant [18]. Infections caused by high-virulence organisms (eg. *S. aureus* and beta-haemolytic Streptococcus spp.) can develop after 7–10 days. It is essential to recognise early PJIs as soon as possible because biofilm is unmature at this stage and therefore it can be eradicated more effectively [16, 17, 19]. Low-grade infections develop six weeks to two years after surgery and are generally caused by low-virulence organisms, eg. *S. epidermidis* and *C. acnes*. Most of the PJIs are recognised in this period. Biofilms are mature at this stage therefore implants cannot be saved, ie. all components must be carefully removed [19, 20]. Acute haematogenous infections may develop any time after surgery [18]. Symptoms develop suddenly without prodromal sings resulting in decreased mobility. Pathogens spread from a different source of infection and reach the implant with the bloodstream. Most frequent sources are skin and soft tissue infections, urinary tract infections, respiratory and gastrointestinal foci [21, 22].

The aim of our study was to determine recovery rates in patients with PJI undergoing DAIR procedure and to investigate the effect of rifampicin resistance and selected patient-related factors on clinical outcome. The overall recovery rate in the rifampicin-sensitive group was 72.3%, whereas it was 76.9% if rifampicin-resistant organism was isolated. This is unexpected and may be due to the low number of resistant isolates in the study population. Various recovery rates have been reported in the literature ranging from 54.2 to 91%, respectively [23–25]. In a previous study, we found recovery rate of 92.5% in the rifampicin-sensitive and 60.0% in the resistant group among patients undergoing two-stage revision due to PJI [26]. This may suggest that rifampicin-resistance has higher impact on recovery rates among patients with two-stage revision as compared to DAIR procedure, however, further investigations are required to confirm these findings.

The prevalence of prosthetic joint infections after knee arthroplasty was found higher among males in a study but the difference was not significant [27]. In our cohort, 59.6% were males in the sensitive and 61.5% in the resistant group. The recovery rate was 72.3% for males and 76.7% for females. We found no statistical evidence of the negative impact of sex on recovery. The vast majority of our patients were in the ≥ 60 years age group: the overall mean age was 68.4 years (standard deviation (SD) = 15.8 years). No relation between age and the prevalence of PJI was found in previous studies [27, 28]. In our study, we may assume that age has a presumable negative impact on recovery rate both in the sensitive and the resistant group.

It has been shown that higher BMI results in higher risk of PJIs [29]. In our cohort, there was only a small difference of BMI between the sensitive and resistant group: 30.1 kg/m^2^ and 30.6 kg/m^2^, respectively. Neither clinically relevant nor statistically significant effect of BMI on recovery rates was observed in either the sensitive or the resistant group. Diabetes mellitus has been confirmed as a significant predisposing factor for PJIs [16, 27, 28]. The prevalence of DM was found to be 5.0% in a study on patients with THR and was associated with higher rates of both surgical and non-surgical site infections [30]. In our cohort, we found higher prevalence: 16.4% of the patients had Type 1 or Type 2 DM. Despite this, there was no significant difference in the recovery rate of diabetic and non-diabetic population, although analysis was limited due to the low number of patients in the rifampicin resistant group. However, our data may suggest a negative impact of diabetes on clinical outcome.

Several medical conditions have been demonstrated to increase the risk of development of PJI, hence it is important to consider them in the management of PJIs. Factors increasing the risk of PJI after implanting primary endoprosthesis include diabetes mellitus, urinary tract infection (UTI), high ASA score and immunosuppression [31, 32]. Another study found that obesity, COPD, excessive ethanol consumption, depression and malignancies can also be predisposing factors [33]. Uncontrolled DM, severe obesity (BMI > 40 kg/m^2^), liver failure, renal insufficiency, smoking, drug abuse, previous prolonged hospitalisation, malnutrition, severe acquired immunodeficiency, posttraumatic arthrosis and inflammatory arthropathy also represent risk factors for periprosthetic infections [34]. The prevalence of PJI was also shown higher among patients receiving intraarticular steroid injection [35].

63.8% of the patients had hypertension in the rifampicin-sensitive and 69.2% in the resistant group. From a different perspective, 62.0% of the patients who recovered had hypertension whereas the prevalence was 82.4% among patients with treatment failure. The prevalence was 58.8% and 60.0% in recovered patients in the sensitive and resistant group, however, we found 76.9% and 100% of prevalence in patients who did not recover. Of note, there were only three patients in the last group. Although a previous study demonstrated that the effect of hypertension is statistically not significant [36], we found 1.3 times lower recovery rates among patients with hypertension. It has been observed that PJIs develop more frequently in patients belonging to ASA group III and IV [37]. In the rifampicin sensitive group, we found 70.9% recovery in patients with ASA II score and 80.0% with ASA III. Recovery rates for ASA II and III patients in the resistant group were 80.0% and 50.0%, respectively. We could not confirm the negative impact of higher ASA scores on recovery rates in the two groups.

Risk scores, such as KLIC and CRIME80 are used to assess the probability of therapeutic failure in patients with PJI [38]. Recovery rates were compared among patients with CRIME80 score ≥ 3 (19 cases). Average score of recovered patients was found higher in the resistant group. Nineteen patients had higher than 4.5 KLIC score. There was no significant different between the average score of recovered patients in the sensitive and the resistant group. 92.31% of the patients had KLIC score in the 2–3.5 range in the resistant group. Although the same range was predominant in the sensitive group, distribution appeared more balanced.

It has previously been demonstrated that the injury of knee or hip joint capsule, previous surgery and trauma significantly increase the risk of development of PJI [39]. We examined whether revision prior to DAIR had impact on recovery rates. Patients who had previous revision showed 66.7%recovery in the rifampicin sensitive and 80.0% in the resistant group, whereas the rates were 74.3% and 75.0% without revision. From a different approach, patients without prior revision had slightly higher recovery rates in the sensitive group, however, recovery rates were moderately higher among patients with previous revision in the resistant group.

Pathogenic spectrum has been changing worldwide. Moreover, there is a general increase in antibiotic resistance rates [1, 40] and in the incidence of polymicrobial infections [41]. The basis of the treatment of PJIs is the removal of biofilm, however, this is only achievable in the first 3–4 weeks of infection. After this period, the implant may have to be removed resulting in a significantly prolonged treatment and healing [42]. The biofilm grows continuously on the implant surface before becoming fully mature after 4–6 weeks. Postoperative exploration, effective washing, exchange of mobile components and retaining fix elements can be satisfactory for unmature biofilms. However, for mature biofilms, the procedure must include the complete removal of the implant [4]. Also, diffusion rate of rifampicin depends on the organism as well as the age of biofilm.

In our study, Staphylococcus spp. was predominant in the rifampicin-sensitive group (18–18 *S. aureus* and CNS isolates) followed by *S. agalactiae, S. dysgalactiae* and 6 *C. acnes*, however, *Enterobacterales* (mostly *E. coli*), *P. aeruginosa* and *E. faecalis* were the most frequent isolates in the resistant group. We observed no development of rifampicin resistance in our cohort and none of the patients had received previous rifampicin treatment. A study in 2017 found that coagulase-negative Staphylococci are the most frequent causative agents of PJIs (30–43%) followed by *S. aureus* (12–23%). Streptococci are the second most frequent pathogens causing PJIs, particularly *S. agalactiae*, *S. pyogenes* and *S. dysgalactiae*. Brucella and Candida species have also been detected, however, no pathogen has been identified in 11% of the cases [43]. Streptococcal PJIs mostly develop as a result of haematogenic infection. A study found that 25% of the specimens grew Streptococcus sp. in relation to PJIs and blood cultures were also positive in 22% of the cases. 22% of the infections were polymicrobial, mostly involving *S. aureus* [44].

PJIs caused by *S. aureus* are characterised by strong inflammatory symptoms soon after implantation indicating early postoperative infection. However, coagulase-negative Staphylococci generally cause late and potentially chronic infection with mature biofilm and less characteristic symptoms. MRSA infection itself is a risk factor resulting in more difficult eradication of PJIs. One study concluded that in case of PJIs caused by low-virulence organisms (eg. coagulase-negative Staphylococci, Cutibaterium and Acinetobacter species) DAIR procedure can be appropriate and result in full recovery, however, this finding should be treated with caution. The incidence of PJIs caused by CNS is worldwide increasing [45]. In our cohort, 33.3% of the patients in the sensitive group had infection caused by coagulase-negative Staphylococci, higher than indicated in the literature, however, no infections due to CNS was found in the resistant group. The prevalence of Enterococci in orthopaedic infections is also on an upward trend [46]. In our cohort, 7.5% of the patients had enterococcal PJI.

Before drawing final conclusions, a few limitations of this study need to be considered. Relatively few patients were included, making statistical analysis and determining significance challenging in certain cases. Our study cohort included patients with PJI affecting different joints, which could also interfere with our results. However, identical therapeutic procedures were performed in all cases, therefore in our opinion, this factor might have only moderate effect on the final conclusions. Also, the surgical technique of DAIR was not exactly the same for all patients as mobile parts were not always exchanged, therefore this variable was also evaluated in our study.

## Conclusions

The majority of the patients had PJI caused by a rifampicin-sensitive organism, however, in our study, we could not find enough evidence to confirm that rifampicin resistance is associated with significantly lower recovery rates. Also, the isolation of rifampicin-sensitive organism was not more frequent among recovered patients and only Gram-positive organisms were observed in the treatment failure group. No development of rifampicin resistance was observed in the study population. The most frequent isolates were Staphylococci in the sensitive and Gram-negative rods in the resistant group.

We found that rifampicin resistance, BMI and sex had no statistically significant impact on recovery rates, although increasing age and diabetes may well have a negative clinical impact on clinical outcome. In conclusion, recognition of microbiological and patient-related factors may help estimate the risk and reduce treatment failure rates after DAIR performed in patients with PJI. Further investigations with larger patient cohort are needed to confirm the effect of rifampicin resistance on recovery rates in patients with PJI undergoing DAIR procedure.

## Data Availability

The datasets generated during and/or analysed during the current study are not yet publicly available as this is the first occasion of publishing these results but are available from the corresponding author on reasonable request.

## Abbreviations

PJI: Prosthetic joint infections
DAIR: Debridement, antibiotics and Implant retention
BMI: Body mass index
CNS: Coagulase-negative Staphylococci
DTT: Difficult-to-treat
SD: Standard deviation
DM: Diabetes mellitus
COPD: Chronic obstructive pulmonary disease
RA: Rheumatoid arthritis
CRF: Chronic renal failure
EUCAST: European Committee on Antimicrobial Susceptibility Testing
IQR: Interquartile range
CRP: C-reactive protein
MRSA: Methicillin-resistant *Staphylococcus aureus*
UTI: Urinary tract infection
